# Growth history of hepatitis C virus among HIV/HCV co-infected patients in Guizhou Province

**DOI:** 10.3389/fgene.2023.1171892

**Published:** 2023-06-06

**Authors:** Xiu-Cheng Yang, Zhang-Ping Hong, Yi Wang, Nan Meng, Yong Hu, Qian-Yu Xiong, Da-Wen Qin, Du Shen, Xing-Lin Yang

**Affiliations:** ^1^ Department of Infectious Disease Control, Aba Center for Disease Control and Prevention, Aba, Sichuan, China; ^2^ Department of Laboratory, Guiyang Medical Center for Public Health, Guiyang, Guizhou, China; ^3^ School of Public Health, The Key Laboratory of Environmental Pollution Monitoring and Disease Control, Ministry of Education, Guizhou Medical University, Guiyang, Guizhou, China

**Keywords:** hepatitis C virus, genotype, phylogeographic analysis, China, Guizhou, Guiyang

## Abstract

**Background:** The evolutionary and epidemiological history and the regional differences of various hepatitis C virus (HCV) genotypes are complex. Our aim was to better understand the molecular epidemiology and evolutionary dynamics of HCV among HIV/HCV co-infected individuals in Guizhou Province. This information could contribute to improve HCV prevention and control strategies in Guizhou and surrounding provinces.

**Methods:** The HCV RNA was extracted from the serum of HIV/HCV co-infected patients, and reverse transcription/nested PCR was performed to amplify nucleotide sequences of the C-E1 region. Then, the successfully amplified sequences were selected for phylogenetic analysis. The available C-E1 region reference sequences from the surrounding provinces of Guizhou (Guangxi, Yunnan, Hunan, and Sichuan) were retrieved in GenBank, and the evolutionary analysis by Bayesian Markov chain Monte Carlo (MCMC) algorithm was performed using BEAST software to reconstruct a phylogeographic tree in order to explore their migration patterns. Finally, the epidemiological history of HCV in the Guizhou region was retraced by reconstructing Bayesian skyline plots (BSPs) after excluding sequences from surrounding provinces.

**Results:** Among 186 HIV/HCV co-infected patients, the C-E1 region sequence was successfully amplified in 177 cases. Phylogenetic analysis classified these sequences into six subtypes: 1a, 1b, 3a, 3b, 6a, and 6n. Among them, subtype 6a was the most dominant strain (*n* = 70), followed by 3b (*n* = 55), 1b (*n* = 31), 3a (*n* = 11), 1a (*n* = 8), and 6n (*n* = 2). By reconstructing the phylogeographic tree, we estimated that the 6a strain in Guizhou mainly originated from Yunnan and Guangxi, while the 3b strain emerged due to transmission from the IDU network in Yunnan. Subtypes 1b, 3a, 3b, and 6a, as the major subtypes of HCV in HIV/HCV co-infected individuals in Guizhou, emerged and later grew more rapidly than the national average. Notably, BSPs of the currently prevalent HCV predominant strain subtype 6a in Guizhou have shown a rapid population growth since 2004. Although the growth rate slowed down around 2010, this growth has continued to date.

**Conclusion:** Overall, despite the improvement and implementation of a series of HCV prevention and control policies and measures, a delayed growth pattern may indicate a unique history of the spread of 6a in Guizhou. Its trend as the dominant strain in Guizhou in recent years may continue to increase slowly over subsequent years.

## 1 Introduction

Hepatitis C virus (HCV) is an enveloped virus that contains a single-stranded positive RNA gene of approximately 9.6 kb in length. HCV belongs to the genus *Hepatovirus* of the family Flaviviridae (Reed and Rice, 2000). Infection with HCV can lead to acute and chronic hepatitis, which may progress to liver cirrhosis and hepatocellular carcinoma if not treated promptly and effectively (Gupta et al., 2022). HCV is divided into seven genotypes with more than 67 subtypes (Smith et al., 2014). Genotypes 1, 2, and 3 are distributed worldwide, while other genotypes are mainly distributed in specific regions, such as genotype 4 in the Middle East and Central Africa, genotype 5 in South Africa, and genotype 6 in South and Southeast Asia (Adinolfi et al., 2001; Pybus et al., 2007; 2009). Different genotypes and genetic subtypes differ in terms of clinical symptoms, treatment response, and epidemiology. HIV and HCV infections have similar transmission routes, high-risk behavioral populations, risk factors, and other factors, thus increasing the risk of HIV/HCV co-infection (Alter, 2002; Cohen et al., 2008). In China, the positive rate of HCV antibodies was 0.60% in HIV-negative patients, while the positive rate was significantly higher in HIV-positive patients. In 2010–2012, a retrospective observational cohort study on the National Free Antiretroviral Treatment Program in China showed that 6,149 (18.2%) of 33,861 HIV-infected patients were co-infected with HCV (Zhang et al., 2014). In addition, the WHO reported that among HIV-infected patients, HCV prevalence was the highest in the injecting drug use population (82.4%) (World Health Organization, 2017). In China, the HIV/HCV co-infection rate is higher among intravenous drug users, with a reported HCV co-infection rate of 99.3% among 138 HIV-1-infected intravenous drug users in Yunnan, China (Zhang et al., 2002). In addition to the health hazards caused by simple infection, co-infection with HIV and HCV can also enhance its transmission in the population and accelerate the clinical process of related diseases through the interaction of pathogens, resulting in more serious disease damage (Fuping et al., 2010; Mabileau et al., 2018). As a result, HCV co-infection has become the most common cause of death among HIV/AIDS patients receiving antiretroviral therapy (Chen et al., 2016).

Currently, bioinformatics technology is developing rapidly, and the gene sequence bank is continuously being enriched based on the theory of joint analysis of human population genetics data and the evolutionary molecular clock method, through the use of Bayesian evolutionary analysis software, such as BEAST, as one of the common methods to infer the epidemic and transmission pattern of viruses (Drummond and Rambaut, 2007). Understanding the genetic variation of HCV plays an important role in many fields. First, determining genotype distribution helps track the transmission of HCV both within specific populations and between different populations (Smith et al., 1997; Mohd Hanafiah et al., 2013; Messina et al., 2015). Second, in clinical practice, identifying the different genotypes of HCV can also guide the choice of clinical antiviral therapy and influence the disease course of patients (Legrand-Abravanel et al., 2009). Finally, the genetic diversity of HCV also provides a theoretical basis for vaccine development. Currently, there is no vaccine to prevent hepatitis C worldwide, but it can be cured with early and effective treatment. This requires precise detection and treatment of hepatitis C.

**FIGURE 1:**
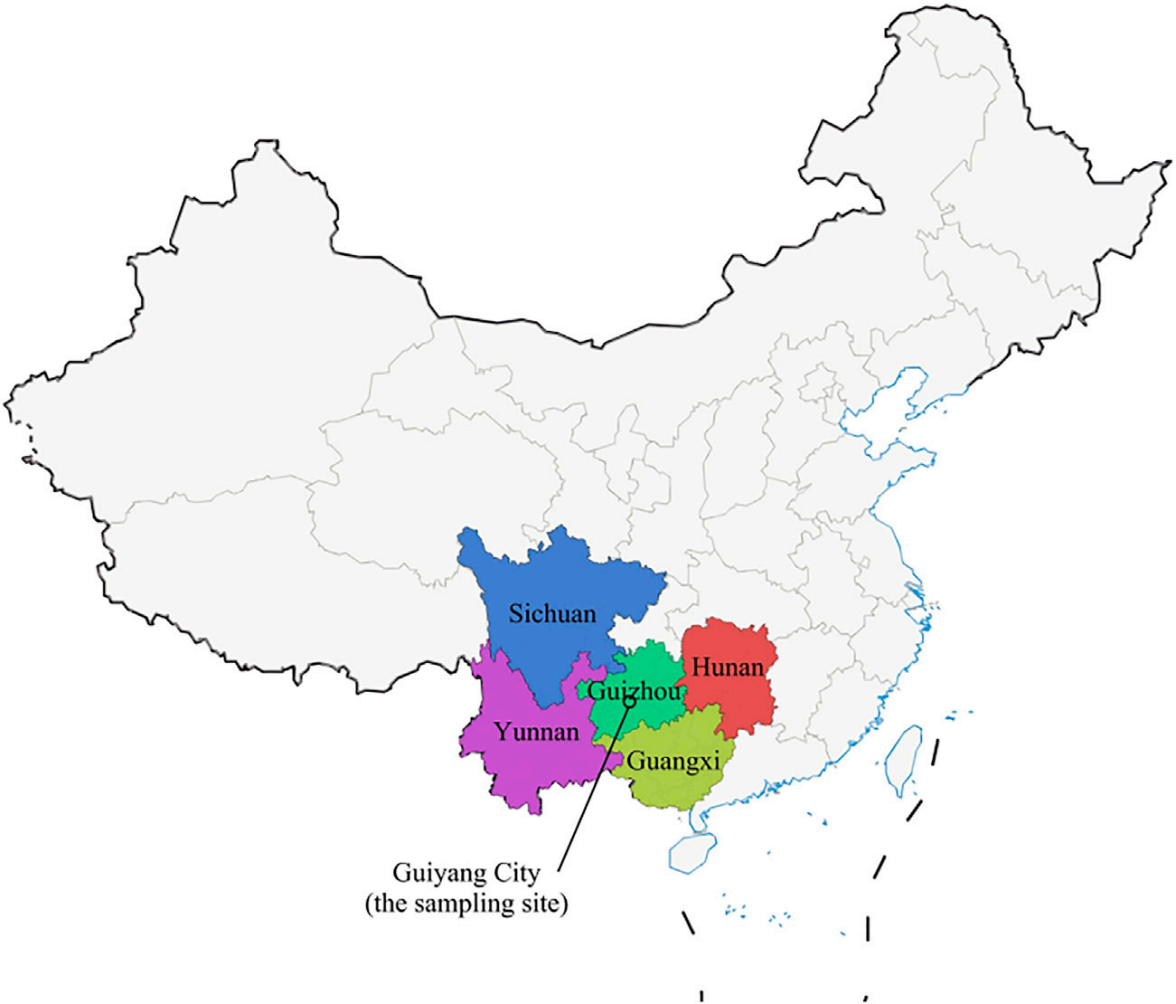
Map of China showing Guizhou Province and its adjacent provinces (Sichuan, Guangxi, Yunnan, and Hunan). The geographic location of Guiyang City, which is our sampling site, is marked.

Guizhou Province, located in southwestern China, has a special geographical location (Zeng et al., 2014), adjacent to two border provinces, Yunnan Province in western China and Guangxi Province in southern China, and Hunan Province in Central China in the east, and bordered by Sichuan and Chongqing to the north, providing favorable conditions for the prevalence and transmission of HIV in Guizhou (Figure 1). Guizhou is a multi-ethnic province, and its capital city Guiyang is a railroad hub in Southwest China, where rapid economic development has led to the spread of disease. A series of previous studies on hepatitis C in China have clarified the molecular epidemiology and evolutionary pattern of HCV in China. However, these studies involved general patients, voluntary blood donors, and injecting drug users (IDUs), and although some patients from Guizhou were included, they were small in number and relatively poorly represented (Huang et al., 2018). In this study, we selected this special population of HIV/HCV co-infection and recruited a larger number of patients with longer consecutive samples, who were patients undergoing follow-up treatment at designated hospitals, to collect more complete information. We aimed to better understand the evolutionary characteristics of HCV genetic subtypes in HIV/HCV co-infected patients in Guizhou Province, which could help improve the prevention and treatment strategies for HCV infection in HIV/HCV co-infected patients in Guizhou Province.

## 2 Materials and methods

### 2.1 Ethical statement

The study was approved by the Institutional Review Board of Guiyang Medical Center for Public Health [Approval Number: 202148]. All participants signed informed consent forms, and the study was performed in strict accordance with the Declaration of Helsinki.

### 2.2 Study design

This study is a cross-sectional study.

### 2.3 Study population and specimen collection

This study selected HIV/HCV co-infected patients who received ART (antiretroviral therapy) at the public health rescue center in Guiyang City, Guizhou Province, during the period 2013–2021. The ART regimen for HIV/HCV co-infected patients was the same as that for HIV alone. As these patients were first diagnosed, the ART regimen was two nucleotide reverse transcriptase inhibitors (NRTIs) combined with class 3 drugs. Class 3 drugs can be non-nucleoside reverse transcriptase inhibitors (NNRTIs) or enhanced protease inhibitors (PIs) or integrase inhibitors (INSTIs) or combined single-tablet regimens (STRs). Once ART is started, patients are treated for life. Inclusion criteria were as follows: 1) age >18 years; 2) positive primary screening and confirmatory tests in all patients; and 3) anti-HCV-positive with HCV RNA quantification in the range of 10^4^–10^7^ IU/mL. Anti-HCV diagnostic kit (Kehua Bio-Engineering, China) and hepatitis C virus RT-PCR kit (Kehua Bio-Engineering) were used. Exclusion criteria were as follows: 1) positive HBV surface antigen (HBsAg) ELISA (Zhongshan Bioengineering, China); 2) previously treated with antiviral drugs (HIV or HCV) treatment; and 3) patients with combined autoimmune hepatitis, malignancy, psychiatric disorders, and other serious diseases. Demographic information (including gender, age, ethnicity, marital status, and transmission route) was collected from the included subjects. A volume of 5 mL of venous blood was drawn before antiviral treatment, and the serum was separated by centrifugation at 3,000 r/min for 10 min, divided into two tubes, and stored at −80°C for freezing.

### 2.4 HCV RNA extraction, PCR amplification, and sequencing

To reduce bias on the amplification of a certain gene subtype due to the primers, the primer sequence was located in the relatively conserved regions of all genotypes of HCV, and the four primers were synthesized according to the nucleotide sequence of the HCV C-E1 region, as described in previous research (Table 1) (Ray et al., 2000). HCV RNA extraction and reverse/nested PCR amplification were performed according to a previous protocol (Yang et al., 2014). Briefly, HCV RNA was extracted using the QIAamp Viral RNA Mini Kit (QIAGEN, Germany), and reverse transcription was performed using the One Step RNA PCR Kit-AMV (Promega, United States). The target fragment of the HCV C-E1 region was 474 bp, which was PCR-amplified using TaKaRa Ex Taq kit (Bao Biological Company, Dalian) on the PCR DNA purification system from Promega. Sequencing was performed at Shanghai Biotech Co., with CEl-ISP as the sequencing primer.

**TABLE 1:** Primer sequence and location of HCV gene subtypes.

Primer	Sequence (5′-3′)	Location*
CEI-ESP	GCA​ACA​GGG​AAC​CTT​CCT​GGT​TGC​TC	834–859
CEI-EAP	CGT​AGG​GGA​CCA​GTT​CAT​CAT​CAT	1 328–1 305
CEI-ISP	AAC​CTT​CCT​GGT​TGC​TCT​TTC​TCT​AT	843–868
CEI-IAP	GTT​CAT​CAT​CAT​ATC​CCA​TGC​CAT	1 316–1 293

Note: *The starting point of this primer is located in the hepatitis C virus strain H77 (GenBank Accession No. NC_004102).

### 2.5 Phylogenetic analysis

As recommended by Simmonds et al. (2005), the following reference sequences of HCV were retrieved from GenBank and included in the analyses: 1a, M62321 and NC_004102; 1b, M58335 and D90208; 2a, AB047639 and D00944; 2b, D10988; 2c, D50409; 3a, D17763 and JN714194; 3b, D49374, JQ065709, and KC844044; 4a, Y11604; 5a, Y13184; 6a, Y12083 and EU246930; 6b, D84262; and 6n, DQ278894 and DQ835768. The 177 C-E1 region amplified sequences from this study were merged with the reference sequences. Sequence alignment of the nucleotide sequence by the MAFFT algorithm was performed using PhyloSuite v1.2.2 (http://phylosuite.jushengwu.com/) (Zhang et al., 2020) software and manually edited. Prior to phylogenetic tree reconstruction, the best-fitting substitution model was selected using the ModelFinder (Kalyaanamoorthy et al., 2017) program on the basis of the Akaike information criterion. The general time-reversible with the invariable-sites-plus-gamma distribution (GTR + I + Γ) substitution model was found to be the best for all resulting sequence datasets. Under this model, the phylogenetic tree was constructed by the maximum likelihood method using MEGA 7.0 (Kumar et al., 2016) software, with which bootstrap analyses were performed in 1,000 replicates. Tree files in the Newick format were generated, and the tree topologies were displayed using the FigTree v1.4.4 to determine HCV subtypes.

### 2.6 Bayesian Markov chain Monte Carlo evolutionary analysis

We retrieved HCV sequences available in the C-E1 region from GenBank (accessed on June 2022) in the neighboring provinces of Guizhou (Yunnan, Guangxi, Hunan, and Sichuan), and based on their GenBank annotations, we selected only viral strains of subtypes 1b, 3a, 3b, and 6a that are predominantly prevalent in Guizhou. The 167 sequences obtained in this study and the aforementioned selected sequences were divided into four datasets according to the same genetic subtypes, and each dataset represented one HCV subtype. We implemented the Bayesian Markov chain Monte Carlo (MCMC) algorithm in the BEAST software package (Drummond and Rambaut, 2007) to track the ancestral relationships and migration patterns of different HCV subtypes in HIV/HCV co-infected patients in Guizhou.

It has been shown that for HCV sequence analyses, the clock models always have exponential models superior to the log-normal and strict molecular clock models (Pybus et al., 2009; Fu et al., 2012; Min et al., 2015; Huang et al., 2018). The demographic models have Bayesian skyline coalescent models that outperform constant size, exponential growth, logical growth, and expansion growth (Fu et al., 2012; Lu et al., 2013; Huang et al., 2018). Therefore, to analyze the different HCV subtypes, we used an exponential clock model combined with the GTR + I + Г substitution model and Bayesian skyline model. We used the rates as described previously (Lu et al., 2013): 1b, 3a, 3b, and 6 *a priori* rates 1.52e-3±2.8325e-5, 1.52e-3±2.8325e-5, 2.737e-3±1.51e-5, and 2.737e-3±1.51e-5, respectively. The MCMC chains were run for 300 million generations, generating a tree every 10,000 generations. The convergence of the parameters was checked using the Tracer v1.7.2 program, and it was determined if the effective sample size (ESS) was greater than 200. We considered that sufficient sampling was achieved when the ESS was ≥200 for all parameters. Maximum clade confidence (MCC) trees were generated by aging 10% of the initially generated trees using TreeAnnotator v1.10.4 software, and the generated posterior trees were viewed using FigTree v1.4.4 software. In this study, we also excluded sequences from the surrounding provinces of Guizhou, combined the same subtype sequences and reference sequences for MCMC algorithm sampling, and used the Tracer v1.7.2 program to reconstruct the Bayesian skyline plot (BSP) to retrace the HCV growth history of HIV/HCV co-infected patients in Guizhou and estimate the evolutionary rate and time of the most recent common ancestor (tMRCA). The BSP is a generalized skyline plot obtained from the target sequence dataset, and then the distribution of the generalized skyline plot is sampled, and these plots are combined to obtain the posterior distribution of effective population size over time. Since the confidence interval of the effective population size at each time point is provided, from the present to the nearest common ancestor of the sampled sequences, the past history of HCV-infected population size can be inferred.

### 2.7 Nucleotide sequence accession numbers

The HCV partial C-E1 nucleotide sequences determined in this study have been deposited in the GenBank sequence database and assigned the accession numbers OQ385213–OQ385389.

### 2.8 Statistical analysis

The database was established in Excel, and statistical analyses were performed using the IBM SPSS V22.0 statistical analysis software package (SPSS Inc., Chicago, IL, United States). In the case of non-normal distributions, descriptive results were summarized using median and interquartile ranges (IQR). Categorical variables were compared using chi-squared tests or Fisher’s exact tests. All tests were two-tailed, and a *p*-value <0.05 was considered statistically significant.

## 3 Results

### 3.1 Patients and samples

A total of 186 HCV RNA seropositive specimens were collected from HIV/HCV co-infected patients during 2013–2021. Excluding amplification failures or unsatisfactory sequences, 177 patients were finally included in this study for analysis. The median age of these patients was 39 years (IQR: 34–44). The male to female ratio was 3.43:1. The basic information of the 177 patients is listed in Table 2.

**TABLE 2:** Characteristics of patients with available HCV C-E1 sequences in HIV/HCV co-infections, 2013–2021, Guizhou, China.

Characteristics	Total sequence (N = 177) (n/N, %)
*Sex*	
Male	137 (77.40)
Female	40 (22.60)
*Age(years)*	
<30	13 (7.35)
30–39	82 (46.33)
40–49	63 (35.59)
≥50	19 (10.73)
*Transmission routes*	
Injecting drug use	70 (39.55)
Heterosexual	57 (32.20)
Men who have sex with men	33 (18.64)
Others	17 (9.61)
*Marital status*	
Unmarried	69 (38.98)
Married	92 (51.98)
Divorced	12 (6.78)
Widowed	4 (2.26)
*Ethnicity*	
Han	137 (77.40)
Miao	13 (7.35)
Buyi	8 (4.52)
Tujia	7 (3.95)
Dong	5 (2.83)
Others	7 (3.95)

Note: “Others” for transmission routes include one case of single plasma collection and 16 cases of unknown. “Others” for ethnicity include three cases of Chuanqing people and one case of each belonging to Bai, Hui, Yao, and Zhuang.

### 3.2 HCV genotyping

**FIGURE 2:**
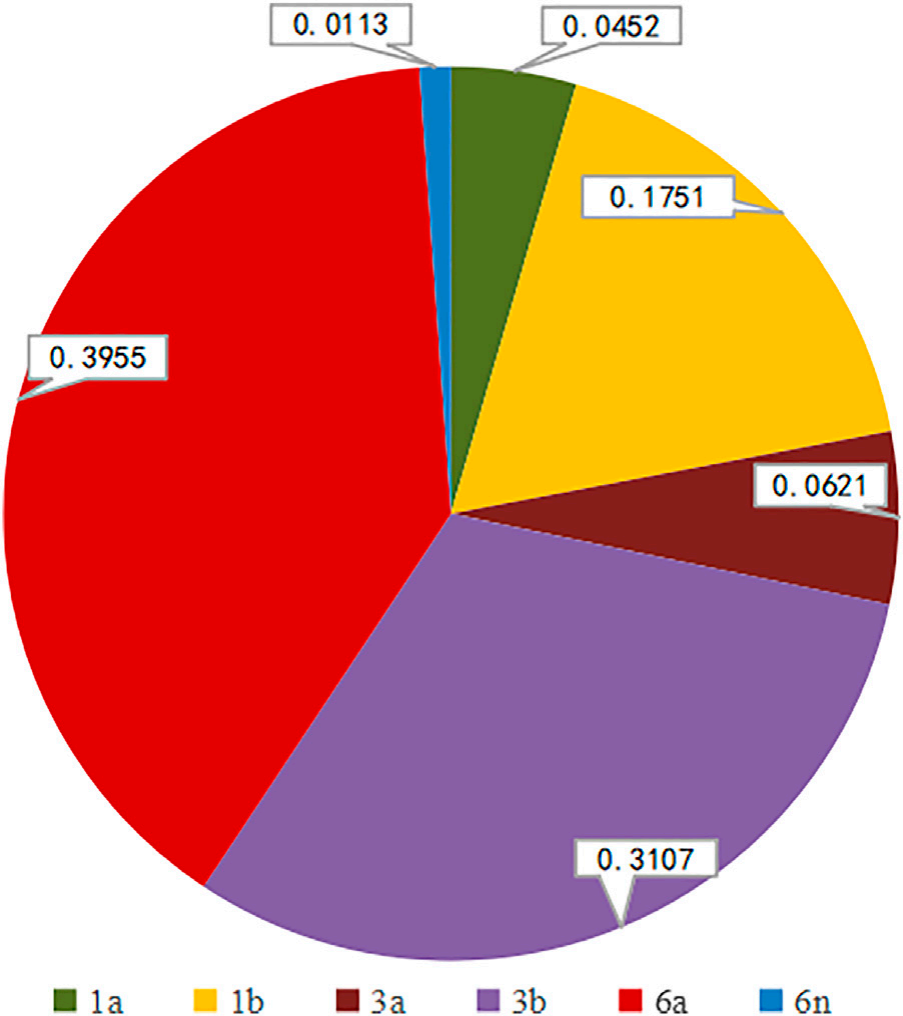
Genotypic distribution of HCV subtypes in core-E1 sequences.

**FIGURE 3:**
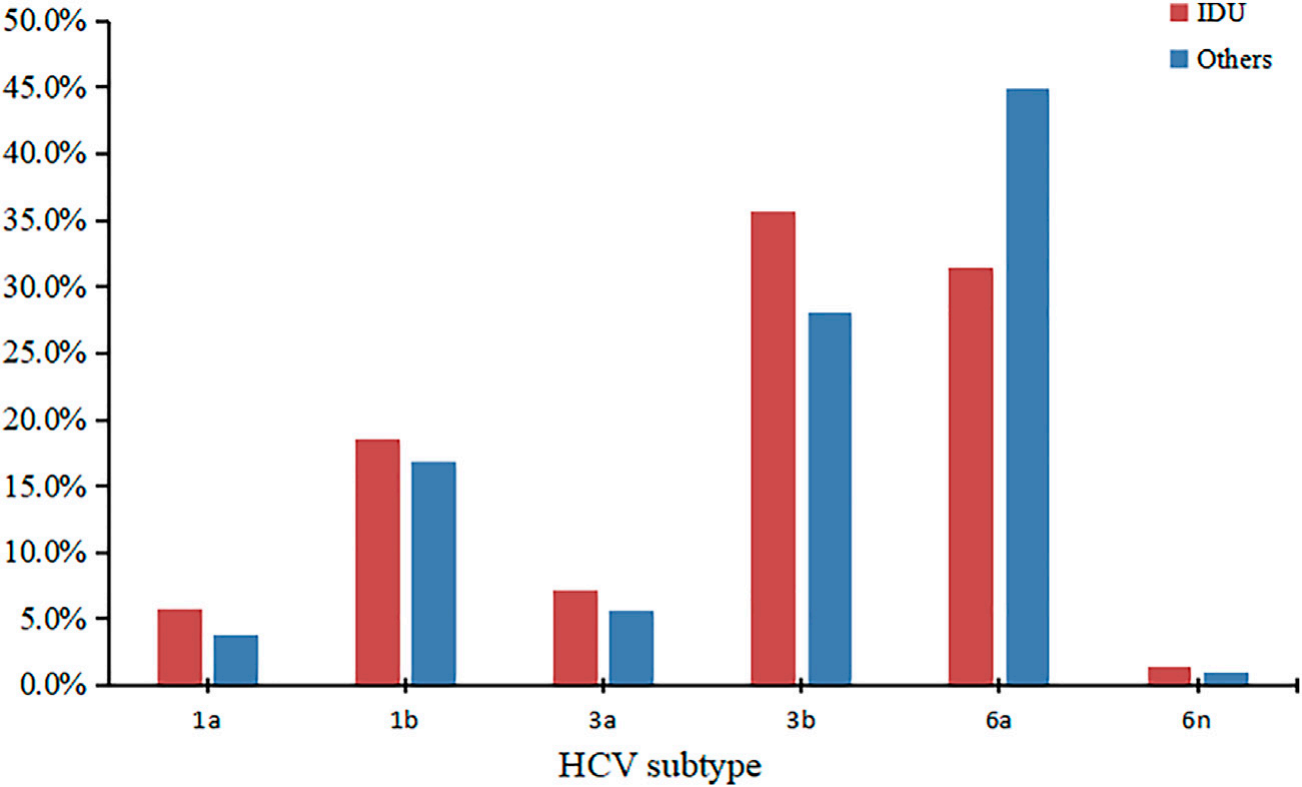
Comparison of the HCV genotype distribution between IDU and others.

We successfully determined the core-E1 region sequences of HCV in 177 co-infected patients. Based on the reference sequences retrieved from GenBank, we classified these sequences into six subtypes: 1a, 1b, 3a, 3b, 6a, and 6n. Among them, subtype 6a was the most dominant (*n* = 70), followed by 3b (*n* = 55), 1b (*n* = 31), 3a (*n* = 11), 1a (*n* = 8), and 6n (*n* = 2). The percentage of each subtype is detailed in Figure 2. The subtypes in recent years are shown in Table 3. There was no significant difference in the constitution of HCV genotypes among HIV/HCV co-infected individuals in different years (*p =* 0.946). According to the route of infection, the 177 HIV/HCV co-infected patients were divided into the intravenous group and the other routes of transmission group (heterosexuality, men who have sex with men, blood transfusion, and other routes). There was no difference in the gene subtype composition ratio between the two groups (*p* > 0.05). For details, see Figure 3.

**TABLE 3:** Distribution of HCV subgenotypes in HIV/HCV co-infection patients in Guizhou, stratified by period, 2013–2021 (*n* = 177).

Year	Number (N = 177)	HCV subgenotypes (n/N, %)						*p*
		1a (*n* = 8)	1b (*n* = 31)	3a (*n* = 11)	3b (*n* = 55)	6a (*n* = 70)	6n (*n* = 2)	
2013–2015	47	3 (6.38)	6 (12.77)	3 (6.38)	17 (36.17)	18 (38.30)	0 (0.00)	0.946
2016–2018	58	2 (3.45)	11 (18.97)	3 (5.17)	20 (34.48)	21 (36.21)	1 (1.72)	
2019–2021	72	3 (4.17)	14 (19.44)	5 (6.94)	18 (25.00)	31 (43.06)	1 (1.39)	

Note: *p* for Fisher’s exact tests.

### 3.3 Phylogenetic analysis

**FIGURE 4:**
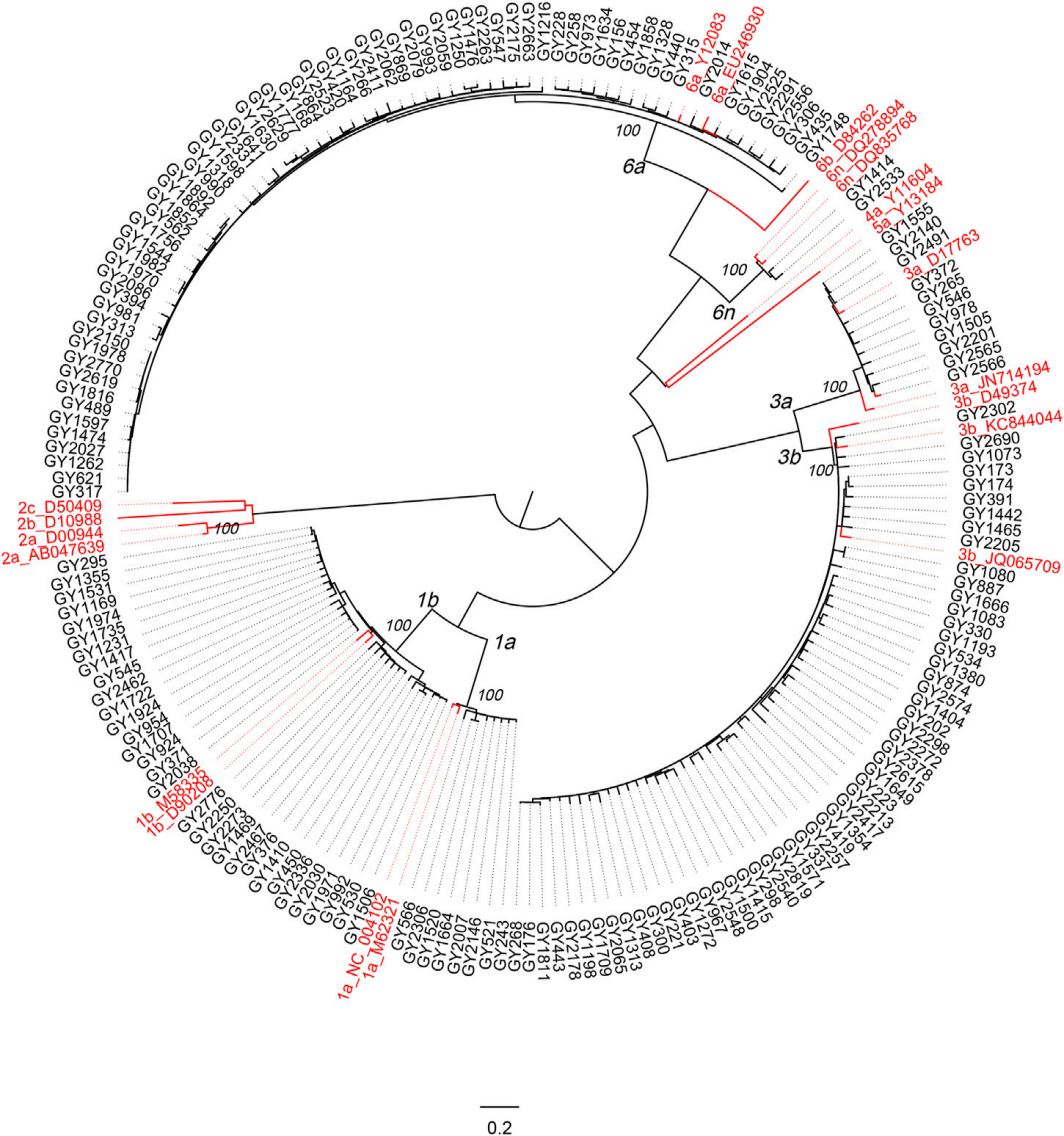
Phylogenetic analysis on the C-E1 gene sequences of HCV from HIV/HCV co-infected patients attending the Guiyang Medical Center for Public Health, Guizhou, China. The sequences from Guiyang patients are colored in black, and reference HCV sequences are colored in red. All references shown in red have the format: subtype_Accession. Otherwise, the isolate ID was used as the label. Full (100%) bootstrap supports are indicated at the most peripheral internal node of each cluster. The horizontal ruler of length 0.2 units per nucleotide per site at the bottom center of the tree acts as a guide to genetic distances.

A circular phylogenetic tree was constructed based on the 177 sequences of the C-E1 region and 20 reference sequences of known genotypes (Figure 4). Significant diversity of HCV isolates in HIV/HCV co-infection patients in Guizhou was shown, and sequences of the same subtypes were closely grouped in the circular tree with full (100%) bootstrap support.

### 3.4 Phylogeographic analysis

To estimate the evolutionary relationships and emergence times of the four major HCV subtypes from HIV/HCV co-infected patients in Guizhou with the surrounding provinces, we performed evolutionary analysis using C-E1 sequences. This included a search from GenBank of 259 reference sequences previously reported in Guizhou representing the four surrounding provinces and 167 viral sequences representing the major HCV subtypes isolated from Guiyang City, Guizhou Province, which was identified in this study.

### 3.5 Subtype 1b

**FIGURE 5:**
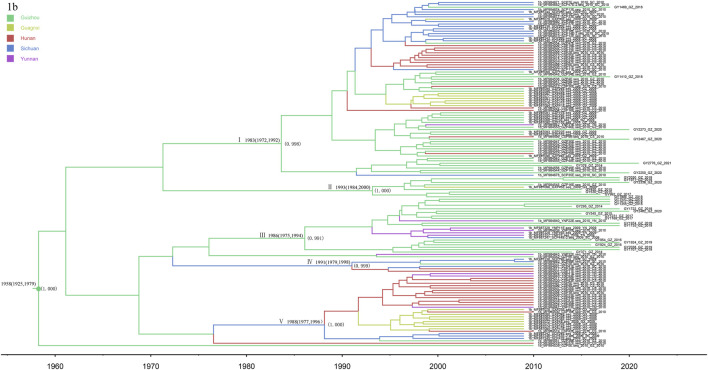
MCC tree based on the C-E1 region of subtype 1b. Branches were colored according to their sampling regions in China, as specified in Figure 1. Time scale runs from 1960 to 2020. Only the posterior values of branches >0.9 are shown.

The phylogeographic tree analysis of subtype 1b showed that the Guizhou isolates were very closely related to isolates from neighboring provinces (Figure 5). We can roughly divide the MCC tree of the 1b sequence into five clusters, where all have a high posterior probability. Most of the isolates from Guizhou were concentrated in clusters I, II, and III, and 31 sequences were newly identified in this study. Clusters VI and V indicate that Sichuan Province may be the origin of the transmission to the other four provinces, spreading subtype 1b from Sichuan to the surrounding provinces. Subtype 1b was found in five provinces, consistent with the national prevalence of subtype 1b nationwide.

### 3.6 Subtype 3a

**FIGURE 6:**
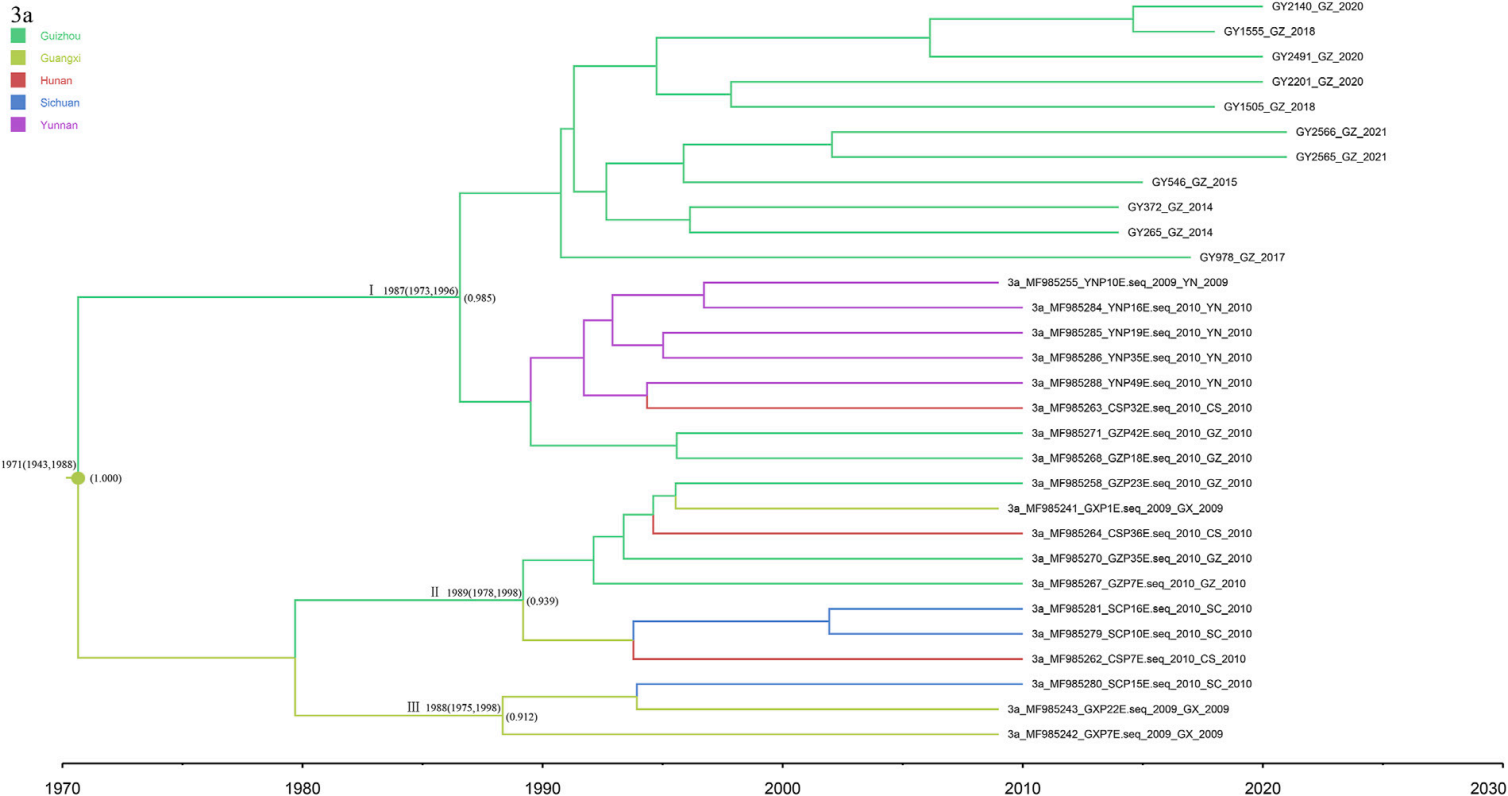
MCC tree based on the C-E1 region of subtype 3a. Branches were colored according to their sampling regions in China, as specified in Figure 1. Time scale runs from 1970 to 2030. Only the posterior values of branches >0.9 are shown.

The 11 newly discovered 3a sequences from this study were analyzed in association with 19 reference sequences (Figure 6). The obtained MCC trees were roughly divided into three clusters, all with high posterior probability. Among them, the 11 newly discovered Guizhou 3a subtypes were all in cluster I, which is clustered with the Yunnan sequence, and we dated their common ancestor to approximately 1987 (95% CI: 1973–1996). Clusters II and III indicate that Guizhou and Guangxi may have played a bridging role in the spread of subtype 3a, which spread successively to Hunan and Sichuan.

### 3.7 Subtype 3b

**FIGURE 7:**
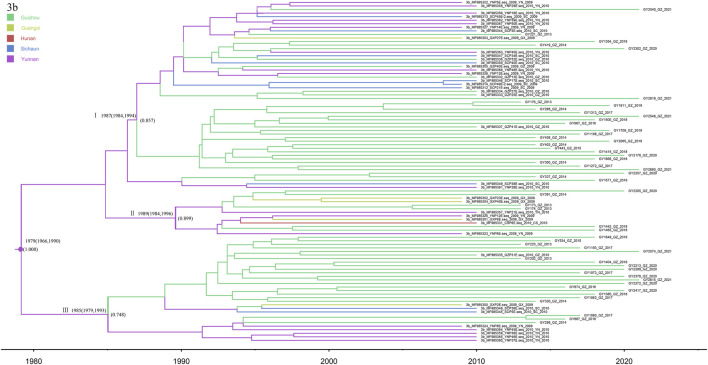
MCC tree based on the C-E1 region of subtype 3b. Branches were colored according to their sampling regions in China, as specified in Figure 1. Time scale runs from 1980 to 2020. Only the posterior values of branches >0.7 are shown.

Notably, subtype 3b was the second most common category of isolates from Guizhou province, detected in 55 patients. We analyzed these sequences together with the 41 reference sequences, allowing us to construct the phylogenetic tree (Figure 7). The phylogeographic tree analysis of subtype 3b indicates that Yunnan Province may be the origin of subtype 3b in Guizhou and its surrounding provinces. We roughly divided the MCC tree of the 3b subtype sequence into three clusters, all of which had a high posterior probability. Most of the isolates from Guizhou were concentrated in clusters I and III. The time scale suggests that multiple 3b strains were introduced into Guizhou Province around 1987, and a high posterior probability suggests a possible origin in Yunnan Province. Subsequently, a larger geographic fusion to neighboring provinces occurred.

### 3.8 Subtype 6a

**FIGURE 8:**
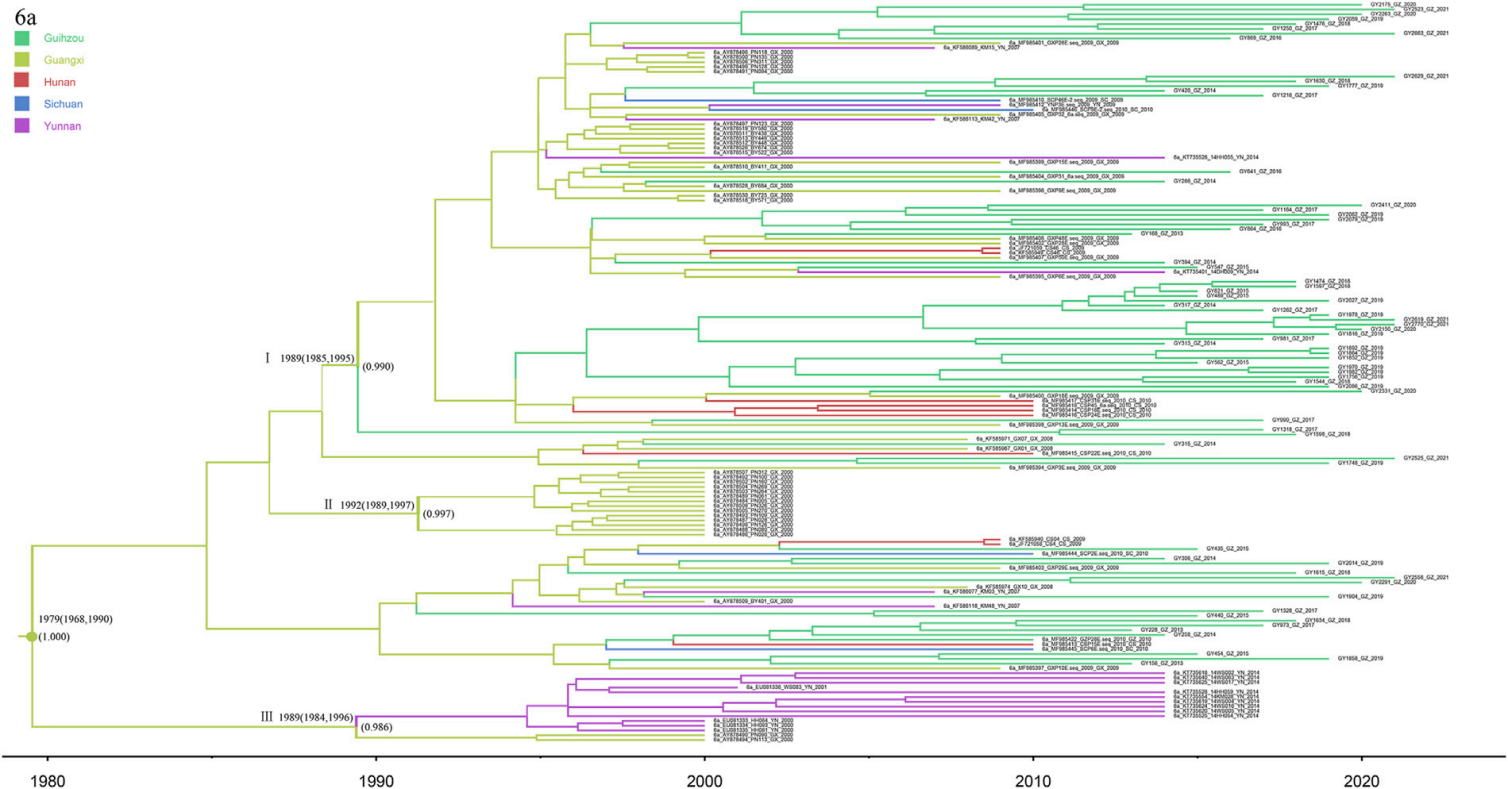
MCC tree based on the C-E1 region of subtype 6a. Branches were colored according to their sampling regions in China, as specified in Figure 1. Time scale runs from 1980 to 2020. Only the posterior values of branches >0.9 are shown.

In this study, subtype 6a was used as the most dominant subtype strain of HCV in HIV/HCV co-infected patients in Guizhou, detected in 70 co-infected patients (Figure 8). The phylogeographic tree was reconstructed by merging with and analyzing 85 reference sequences from Guangxi, Yunnan, Sichuan, and Hunan to explore the origin of subtype 6a in Guizhou. The three 6a clusters shown in the tree all have high posterior probabilities. The Guangxi and Yunnan strains were placed at the bottom, and cluster III contained the most direct descendants of the earliest 6a subtype common ancestor in this study sequence, which started to differentiate around 1979 (95% CI: 1968–1990), and it is considered to be the origin of the 6a strains in the southwestern part of China. Cluster I can account for the transmission migration of 6a from Guangxi to Guizhou, Hunan, Sichuan, and Yunnan provinces. Cluster Ⅱ were all Guangxi sequences, and their divergence dates back to 1992 (95% CI: 1989–1997), both later than clusters Ⅰ and Ⅱ.

### 3.9 Bayesian skyline plot analysis

**FIGURE 9:**
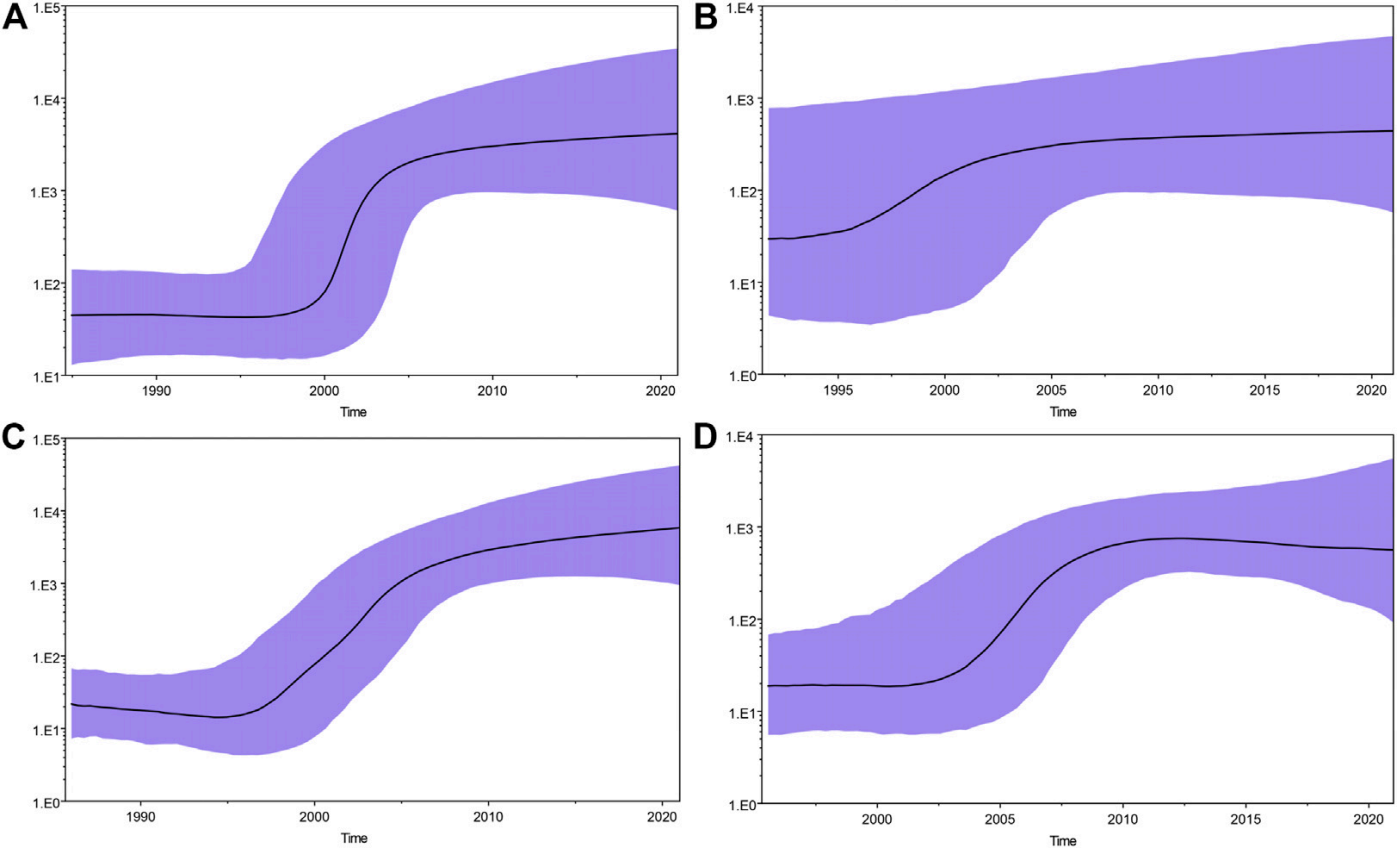
Bayesian skyline plots for four major HCV subtypes in Guizhou. **(A)**, **(B)**, **(C)**, and **(D)** represent HCV genotypes 1b, 3a, 3b, and 6a, respectively. The black curve represents the effective population size through time. The upper and lower purple curve areas define the 95% highest posterior density confidence interval. A vertical ruler on the left (*Y*-axis) measures the effective population size, while a horizontal scale (*X*-axis) at the bottom measures time. The population size of subtypes 1b, 3a, and 3b appear to be relatively constant from approximately 2005 to present. However, the population size of subtype 6a appears to be relatively constant from approximately 2010. The rapid growth periods for the four major HCV subtypes were found to be different: 2000–2005 for 1b, 1995–2005 for 3a, 1996–2006 for 3b, and 2004–2010 for 6a.

After excluding the sequences of the surrounding provinces of Guizhou, we also performed Bayesian skyline plot analysis to estimate the tMRCA (time of most recent common ancestor) and growth dynamics of these four major HCV subtypes (Figure 9). The rapid growth time of subtype 6a lagged behind 1b, 3a, and 3b, and an exponential growth trend of subtype 6a was observed only after the subtypes of HCV genes in these three reached a steady rise. The tMRCA was 1957 (95% CI: 1903–1985) for subtype 1b, 1976 (95% CI: 1951–1992) for 3a, 1988 (95% CI: 1978–1997) for 3b, and 1992 (95% CI: 1982–2001) for 6a. Using Tracer software, we plotted Bayesian skyline plots to view the rapid phase of viral population growth. Subtypes 1b, 3a, 3b, and 6a appeared in this phase during 2000–2005, 1995–2005, 1996–2006, and 2004–2010, respectively. Before the rapid phase, 1b had maintained a relatively stable population size over the decade, while 3a, 3b, and 6a each had passed a slow growth over many years. Following the rapid growth phase, the four subtypes still showed an upward trend to date, although at a much slower rate.

## 4 Discussion

We selected serum samples from 186 HIV/HCV co-infected patients in Guiyang, Guizhou Province, and successfully sequenced the C-E1 region of HCV in a total of 177 co-infected patients. Subtypes 1b, 3a, 3b, and 6a were the main subtypes of HCV in HIV/HCV co-infected patients in Guizhou, which was basically consistent with the genetic subtype results of HCV patients in our previous study in Guizhou (Yang et al., 2014). This is also generally consistent with the proportion of HCV genotypes among HIV/HCV co-infected patients reported in Guangdong in recent years (Deng et al., 2022). However, the difference was that subtype 1b was the dominant subtype in the previous study, whereas subtype 6a was the dominant subtype in HIV/HCV co-infected patients in this study, which may be due to the difference in sample composition. In this study, it was also found that the distribution of subtypes in different years did not change, suggesting that subtype 6a, which is currently the predominant subtype, may continue to be widely transmitted among HIV/HCV co-infected patients.

In this study, subtype 1b was found to be the earliest epidemic strain of HCV subtypes in Guizhou Province (1957: 1903–1985). Subtype 1b grew rapidly from 2000 to 2005 and slowly from 2005 to present. Its emergence and rapid growth were later than the national average (Huang et al., 2018), which is related to relatively slow transportation and economic development of Guizhou Province, which is located in the hinterland of Southwest China. The recently discovered subtype 1b in Guizhou is mainly concentrated in clusters I, II, and III. In addition, clusters VI and V indicate that Sichuan Province, as the origin of the other four provinces, spread subtype 1b from Sichuan to the surrounding provinces. In China, the main epidemic subtype of HCV is 1b, accounting for approximately 73% of all HCV infections (Zhang et al., 2011). In this study, the Guizhou isolates were closely related to those from neighboring provinces, indicating that subtype 1b has spread widely throughout the country, with geographic convergence occurring among the provinces.

The most recent divergence of subtype 3a in Guizhou was in 1976 (95% CI: 1951–1992), and a rapid growth trend occurred from 1995 to 2005. The MCC tree showed that Guizhou and Guangxi may have played a bridging role in the spread of subtype 3a, which spread successively to Hunan and Sichuan. The tree suggested that the 3a strain in Guizhou Province may have originated in Guangxi, which is the southern border province of China, and spread through the IDU network due to drug smuggling (Garten et al., 2004).

In this study, the most recent divergence of subtype 3b in Guizhou was in 1989 (95% CI: 1978–1997), which showed a rapid growth trend from 1996 to 2006. The MCC tree indicated that subtype 3b probably originated in Yunnan Province and migrated to Guizhou and its surrounding provinces. According to previous studies, subtype 3b is more common among IDUs than the general population (Garten et al., 2005; Min et al., 2015; Wang et al., 2019). Therefore, we hypothesize that its migration from Yunnan Province to other regions was mainly spread through the IDU network. Yunnan Province is located in the southwestern border; its Honghe Prefecture has a border with Vietnam and is close to the Golden Triangle that connects Southeast Asian countries, where intravenous drug use is more serious and HIV/HCV infection rates are relatively high nationwide (Ma et al., 2019). This is consistent with Yunnan’s close proximity to the Golden Triangle and as a drug smuggling route in China (Xia et al., 2008).

Subtype 6a was the most dominant viral strain of HCV genotypes among HIV/HCV co-infected patients in Guizhou Province in this study. It is also the most recent newly identified epidemic case of HCV subtypes in Guizhou Province (1993: 1982–2001). From 2004 to 2010, subtype 6a grew rapidly, followed by a slow growth from 2010 to present. According to previous studies, Vietnam is the origin of subtype 6a in China, from where it was first spread to Yunnan and Guangxi provinces. Then, it spread to Guangdong Province and became the predominant subtype of HCV prevalent in Guangdong, and it later migrated to other provinces (Fu et al., 2012). The phylogeographic tree of subtype 6a in this study showed a trend of migration from Guangxi to Guizhou and surrounding provinces. As with subtype 3b, transmission through the IDU network may have played a key role in the early introduction of subtype 6a from Vietnam to the southwestern provinces of China (Fu et al., 2012) and then to Guizhou Province. In fact, the tree shows the recent 6a migration trend in Guizhou since only one of the sequences we studied was an early study sequence in Guizhou. In addition, there was no difference in the composition ratio of subtype 6a among HIV/HCV co-infected patients with IDU transmission and patients with other routes of transmission in Guizhou in recent years. Therefore, subtype 6a in Guizhou cannot be adequately explained by IDU network transmission. At present, Guizhou, as the first province in western China to achieve county-to-county high-speed access, entered the “high-speed railway era” in 2014, which is the transportation hub of Southwest China. Guizhou also has the country’s first national comprehensive pilot zone for big data, a world-renowned mountain tourism destination, which has become a major part of the Yangtze River Economic Belt. The rapid economic development has attracted laborers and tourists from all over the country, and the flow of people between provinces has become more frequent and convenient, allowing subtype 6a to appear widely spread in Guizhou.

IDU and blood are considered the most important transmission routes of HCV (Simmonds, 2004). The percentage of HIV/HCV co-infected patients who contracted HCV through intravenous injection and sexual transmission in this study was as high as 71.75%, suggesting that this group is still a priority target for prevention and treatment. With the implementation of China’s economic reform and opening up in 1979, various subtypes of HCV became prevalent in China and developed rapidly during China’s market economy boom in the 1990s (Huang et al., 2018). However, Guizhou’s economy developed at a later pace than other Chinese provinces, thus exhibiting similar later growth trends across subtypes to other provinces. From approximately 2005 to date, the growth of subtypes 1b, 3a, and 3b has stabilized, while the growth of subtype 6a has slowed down and stabilized from 2010 to present. This is closely related to the implementation of the following health regulations and policies in China: 1) to further improve sterilization and isolation and eliminate the medical transmission of hepatitis and other diseases, the Ministry of Health issued a “Notice on promoting the use of disposable plastic syringes, infusions, blood tubes, and needles” in 1987, and in 1992, it issued the “Notice on strengthening the management of clinical use of single-use infusion (blood) sets and single-use sterile syringes.” 2) In 1998, China promulgated the Blood Donation Law, and in 2001, the General Administration of Quality Supervision, Inspection, and Quarantine issued the “Requirements for Health Examination of Blood Donors” to provide technical guidance for blood donation work in general blood stations. 3) To strengthen the quality and safety management of blood purification and to ensure medical quality and patient safety, the Ministry of Health issued the “Standard Operating Procedures for Blood Purification” in 2010. 4) Since 1992, a theme slogan has been determined for the International Drug Control Day each year to achieve international attention and joint participation, and China also implemented the Anti-Drug Law of the People’s Republic of China in 2008. 5) To achieve the World Health Organization’s goal of eliminating the public health hazards of viral hepatitis by 2030, China formulated the Work Program for Action to Eliminate Hepatitis C Public Health Hazards (2021–2030) in 2021. The implementation of these health policies has been effective in controlling the rapid growth of HCV, particularly through the blood route. In addition, we are getting closer to the goal of eliminating hepatitis C by 2030 through continuous improvement of health policies and the development of additional and more effective measures.

## 5 Strengths and limitations

To the best of our knowledge, this study is an epidemiological and evolutionary analysis of HCV genotypes in a larger sample of HIV/HCV co-infected patients in Guizhou Province. Compared with previous studies, the study showed strengths in the following three areas: first, the sequences analyzed represent the HCV virus strains currently prevalent in HIV/HCV co-infected patients in Guizhou Province. HIV patients are provided with free medication in China and are regularly followed up by designated hospitals. The Guiyang Public Health and Treatment Center is also the designated hospital for AIDS treatment in Guiyang City, Guizhou Province. Therefore, the samples from this hospital can better represent HIV/HCV co-infected patients in Guiyang City. Second, in this study, all samples were obtained from HIV/HCV co-infected patients before ART was administered. The results of this study are more representative of the history of HCV development in a high-risk population of HIV/HCV co-infected individuals than those of the previous studies using samples from injecting drug users, blood donors, or patients with chronic liver disease. Third, we estimated not only the evolutionary relationship between the origin of HCV genotypes in Guizhou Province and the four surrounding provinces but also for the first time the possible emergence time of major HCV subtypes in a special population of HIV/HCV co-infected patients in Guizhou Province after excluding external sequences. We also estimated the rapid growth period of the four major HCV subtypes by BSP and reconstructed their epidemiological history. However, there are some limitations to our study. This study did not include HCV-simple infected patients for differential analysis and was not comprehensive. In addition, due to financial constraints, we selected the C-E1 region for amplification and sequence analysis of the isolates. We hope to include the NS5B region for accurate sequence evolution analysis in the future.

## 6 Conclusion

Overall, despite the improvement and implementation of a series of HCV prevention and control policies and measures, a delayed growth pattern may indicate a unique history of the spread of subtype 6a in Guizhou. Its trend as the dominant strain in Guizhou in recent years may continue to increase slowly over time.

## Data Availability

The datasets presented in this study can be found in online repositories. The names of the repository/repositories and accession number(s) can be found below: https://www.ncbi.nlm.nih.gov/, OQ385213–OQ385389.
